# Characteristics and Risk Factors of Delayed Perforation in Endoscopic Submucosal Dissection for Early Gastric Cancer

**DOI:** 10.3390/jcm13051317

**Published:** 2024-02-26

**Authors:** Taro Akashi, Naoyuki Yamaguchi, Junya Shiota, Maiko Tabuchi, Moto Kitayama, Keiichi Hashiguchi, Kayoko Matsushima, Yuko Akazawa, Kazuhiko Nakao

**Affiliations:** 1 Department of Gastroenterology and Hepatology, Nagasaki University Graduate School of Biomedical Sciences, Nagasaki 852-8501, Japan; jshiota@nagasaki-u.ac.jp (J.S.); mokitayama@nagasaki-u.ac.jp (M.K.); khashiguchi@nagasaki-u.ac.jp (K.H.); kazuhiko@nagasaki-u.ac.jp (K.N.); 2 Department of Endoscopy, Nagasaki University Hospital, Nagasaki 852-8501, Japan; naoyuki3334@hotmail.com; 3 Department of Histology and Cell Biology, Nagasaki University Graduate School of Biomedical Sciences, Nagasaki 852-8523, Japan; m.tabuchi@nagasaki-u.ac.jp (M.T.); akazaway@nagasaki-u.ac.jp (Y.A.); 4 Medical Education Development Center, Nagasaki University Hospital, Nagasaki 852-8501, Japan; kmatsu@nagasaki-u.ac.jp

**Keywords:** delayed perforation, endoscopic submucosal dissection, early gastric cancer, postoperative stomach, histopathological ulceration

## Abstract

(1) **Background**: Delayed perforation after gastric endoscopic submucosal dissection (ESD) for early gastric cancer is a relatively uncommon and serious complication that sometimes requires emergency surgery. This study aimed to determine the clinicopathological features, risk factors, and appropriate management strategies for delayed perforation. (2) **Methods**: This study included 735 patients with 791 lesions who underwent ESD for early gastric cancer at a single institution between July 2009 and June 2019. We retrospectively compared the clinical features of patients with and without delayed perforations. (3) **Results**: The incidence of delayed perforations was 0.91%. The identified risk factors included a postoperative stomach condition and histopathological ulceration. A comparison between delayed and intraoperative perforations revealed a postoperative stomach condition as a characteristic risk factor for delayed perforation. Patients with delayed perforation who avoided emergency surgery tended to exhibit an earlier onset of symptoms such as abdominal pain and fever. No peritoneal seeding following delayed perforation was observed for any patient. (4) **Conclusions**: A postoperative stomach condition and histopathological ulceration were risk factors for delayed perforation. Delayed perforation is a significant complication that requires careful monitoring after gastric ESD for early gastric cancer, particularly in patients with postoperative gastric conditions.

## 1. Introduction

Endoscopic submucosal dissection (ESD) has emerged as a widely adopted, minimally invasive treatment for early gastric cancer, particularly in patients with a low risk of lymph node metastasis (LNM) [1]. However, the successful implementation of gastric ESD requires the ability to accurately diagnose an early gastric cancer that is suitable for the surgery along with delicate endoscopic manipulation, which demands advanced skills and experience. Furthermore, various complications with gastric ESD have been reported, with some being serious enough to necessitate surgical intervention. The operator performing gastric ESD must be well versed in recognizing these complications and possess the ability to respond effectively in the event of their occurrence. The complications associated with gastric ESD include bleeding, perforation, and stenosis, with bleeding divided into intraoperative and postoperative bleeding and perforation into intraoperative and delayed perforation. The reported rates are 4.4% for postoperative bleeding, 2.3% for intraoperative perforation, 0.4% for delayed perforation, and 1.9~2.5% for stenosis [2,3,4]. The incidence of delayed perforation after gastric ESD is very low compared with that of other complications. Due to the rarity of delayed perforation after gastric ESD, research on this complication is limited. Therefore, much remains unknown regarding the clinical characteristics, risk factors, and effective management of delayed perforations. However, intraoperative perforation has a higher incidence than delayed perforation. Numerous studies have examined intraoperative perforations. The risk factors for intraoperative perforation include a tumor diameter exceeding 20 mm, lesions in the U-region, the presence of ulcer scars, and lesions on the greater curvature [5,6,7]. Emergency surgery was once required for intraoperative perforation during gastric ESD. However, recent advancements such as the successful clip closure of intraoperative perforations have reduced the need for immediate surgical interventions [8]. Despite these advancements, delayed perforation continues to be a significant concern because of its potential to necessitate emergency surgery [9]. Delayed perforation often occurs within a few days after ESD, although it has been reported to occur 24 days after the procedure [10]. Therefore, it is difficult to predict when a delayed perforation may occur, and it is unclear what should be carefully monitored and managed after ESD. This study aimed to delineate the clinical characteristics, risk factors, and effective management of delayed perforations during gastric ESD for early gastric cancer.

## 2. Materials and Methods

### 2.1. Patients

**Figure 1:**
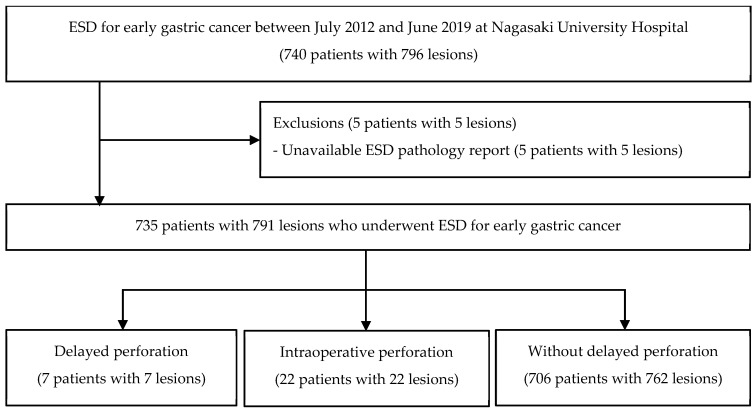
Flowchart of study patients. Abbreviations: ESD, endoscopic submucosal dissection.

This study included 796 lesions in 740 patients who underwent gastric ESD for early gastric cancer at Nagasaki University Hospital between July 2012 and June 2019. Patients with insufficient post-ESD pathology reports (five patients with five lesions) were excluded. This study focused on delayed perforations, particularly those that did not involve intraoperative perforations. Delayed perforation was observed for 7 patients with 7 lesions, intraoperative perforation for 22 patients with 22 lesions, and no delayed perforation for 706 patients with 762 lesions. These cases were subsequently analyzed (Figure 1). Additionally, an analysis was conducted on five patients with five lesions who experienced delayed perforation but did not require surgery along with two patients with two lesions who underwent surgery.

### 2.2. ESD Procedures

Early gastric cancer ESDs were performed in accordance with the Japanese guidelines for gastric cancer treatment [11]. En bloc resection was defined as an intact resection of the lesion rather than in segments. En bloc complete resection was defined as en bloc resection with clear margins (no tumor on horizontal or vertical edges) meeting the following criteria: (1) tumor size ≤ 2 cm, differentiated type intramucosal cancer, no ulcer; (2) tumor size > 2 cm, differentiated type intramucosal cancer, no ulcer; (3) tumor size ≤ 3 cm, differentiated type intramucosal cancer, with ulcer; (4) tumor size ≤ 2 cm, undifferentiated type intramucosal cancer, no ulcer; (5) tumor size ≤ 3 cm, differentiated type with submucosal invasion (SM < 500 μm). Curative resection was defined as a complete en bloc resection with no vascular invasion [11]. ESD was performed as previously described [12]. Gastric ESD was performed using a GIF-Q260J (Olympus Corp., Tokyo, Japan) and a high-frequency electrosurgical unit VIO300D (ERBE, Tubingen, Germany). The electrosurgical knives used for gastric ESD included an insulation-tipped diathermic knife2 (IT-knife2) (Olympus Corp., Tokyo, Japan), Flush knife BT-S (2.0 mm) (Fujifilm Co., Tokyo, Japan), and Dual knife (Olympus Corp., Tokyo, Japan). ESD was performed as follows: First, the lesion was identified and marked with a dotted pattern around its perimeter by using an ESD electrosurgical knife. Next, a 0.4% sodium hyaluronic acid solution (Boston Scientific, Tokyo, Japan) containing indigo carmine, adrenaline, and concentrated glycerin was injected into the submucosal layer, causing it to distend and facilitate mucosal incision and submucosal dissection. The mucosa around the lesion was then incised using an ESD electrosurgical knife, and the submucosal layer beneath the lesion was dissected to remove the lesion. Intraoperative bleeding was controlled using Coagrasper (Olympus Corp., Tokyo, Japan). In some cases, dental floss clip traction was employed [13]. Second-look endoscopy was routinely performed on the day after ESD. If bleeding or exposed vessels were observed at the ulcer base during second-look endoscopy, hemostasis was achieved using coagulation forceps.

### 2.3. Definition of Delayed Perforation

Delayed perforation was defined as a gastric perforation characterized by symptoms of peritonitis or mediastinitis occurring after ESD and were not identified at the conclusion of ESD. Gastric perforation was diagnosed when it was confirmed by endoscopy or computed tomography (CT), which revealed free air or fluid collection.

### 2.4. Evaluation of Clinical Pathological Features, Risk Factors for Delayed Perforation, and Optimal Management

We retrospectively analyzed characteristics specific to delayed perforation by comparing the clinicopathological features of patients with and without delayed perforations. We also compared risk factors for delayed perforation with those for intraoperative perforation. Furthermore, we discussed appropriate management strategies by examining the clinicopathological characteristics of cases in which delayed perforation necessitated surgery versus those in which surgery was not required.

### 2.5. Statistical Analysis

Statistical analyses were conducted using the JMP Pro 17.0 (SAS Institute Japan Co. Ltd., Tokyo, Japan). Fisher’s exact test was used for comparisons between categorical data, whereas the Wilcoxon rank-sum test was used for continuous data comparisons. Logistic regression analysis was used for multivariate analysis, and statistical significance was set at *p* < 0.05.

## 3. Results

Of the 769 early gastric cancers subjected to ESD, delayed perforation occurred in seven lesions, representing a rate of 0.91%. The clinical characteristics of patients with delayed perforation are presented in Table 1.

There were seven instances of delayed perforation, with a median patient age of 74 years. Of these, six patients (85.7%) were male, and the lesions were predominantly located in the lower third of the stomach (six lesions, 85.7%). Two patients underwent gastric surgeries. The curative resection rate was 71.4% (five of seven patients). The median resection size was 42 mm and the median tumor size was 17 mm. Histopathological ulcerations were observed for four lesions (57.1%). Second-look endoscopy was performed on three patients, and one patient experienced postoperative bleeding before the onset of delayed perforation. Computed tomography (CT) was conducted on all patients, confirming the presence of free air in six patients (85.7%) and fluid collection in two patients (28.5%). Symptoms included abdominal pain in six patients (85.7%), fever in one patient (14.3%), and symptoms in six patients (85.7%). The median time to symptom onset for delayed perforation was 14.4 h, and the median time to diagnosis was 23 h. No peritoneal seeding following delayed perforation was observed for any patient (median follow-up, 49 months, with a range of 4–87 months). Among the cases of delayed perforation, emergency surgery was performed on five patients, while two were managed non-surgically.

The clinical characteristics of the patients with and without delayed perforation are shown in Table 2.

When comparing patients with and without delayed perforation, those with delayed perforation had significantly higher rates of en bloc complete resection, postoperative stomach conditions, and histopathological ulcerations (*p* = 0.043, 0.016, and 0.026, respectively). In the multivariate analysis, postoperative stomach conditions and histopathologic ulceration were identified as risk factors for delayed perforation (postoperative stomach: OR = 23.1, 95% CI 3.59–148.64, *p* = 0.001; ulceration: OR = 8.37, 95% CI 1.64–42.7, *p* = 0.016) (Table 3).

Although there was no significant difference in the odds ratio between patients with delayed perforation and those with intraoperative perforation, a trend towards a higher percentage of postoperative stomach conditions was observed in patients with delayed perforation (Table S1).

The clinical characteristics of patients with delayed perforation who underwent surgery and those who did not are shown in Table S2. Although not statistically significant, the trend indicated that patients who avoided surgery developed symptoms earlier than those who avoided surgery (*p* = 0.081).

## 4. Discussion

Delayed perforation following gastric ESD typically occurs 1–2 days after the procedure [14] at a reported rate of 0.4% [2]. Despite its rarity, delayed perforation is a serious complication that often necessitates emergency surgery [9]. Kato et al. [15] reported that delayed perforation occurred in 2 out of 468 patients (0.43%) with gastric noninvasive neoplasia who underwent ESD, and both patients required emergency surgery. Additionally, Suzuki et al. [16] reported that delayed perforation occurred in 7 (0.1%) of 4820 patients with early gastric cancer who underwent ESD. Among them, three (43%) required emergency surgery, while the remaining four were conservatively managed without surgical intervention. The primary causes of delayed perforation are tissue ischemia and necrosis due to excessive energization of the muscle layer [9]. Although intraoperative perforations are usually small in diameter, delayed perforations often present themselves as relatively large defects resulting from extensive muscle layer loss. Some reports suggest endoscopic suturing or conservative treatment as an alternative to surgery [16,17,18]. It has been reported that a perforation size of less than 1 cm is significantly associated with successful nonsurgical treatment of delayed perforation and that endoscopic suturing should be considered first if the perforation size is less than 1 cm [19]. However, the site of delayed perforation and the surrounding mucosa are often fragile, and endoscopic suturing may not be successful. Recently, the efficacy of polyglycolic acid (PGA) sheets (Gunze Co., Osaka, Japan) for delayed perforation after gastric ESD has been reported [20]. Ono et al. covered the delayed perforation site with a PGA sheet cut into strips, applied fibrin glue to the PGA sheet, and secured the PGA sheet to the periulcer mucosa using endoclips. Eleven days after covering the perforation site with a PGA sheet, the sheet spontaneously detached, confirming the complete closure of the perforation site. Although the closure of a delayed perforation is useful, there are cases in which it is better not to close the perforation site. Nagae et al. [17] reported a case in which a delayed perforation site following gastric ESD with a perigastric abscess was treated conservatively without closure. They concluded that if the perforation site is not large and an encapsulated perigastric abscess is identified, the conservative treatment period may be shortened by leaving the site open and expecting spontaneous drainage of pus from the perforation site. When a delayed perforation occurs, it is crucial not only to detect free air on a CT scan but also to recognize perigastric fluid collection to determine the appropriate treatment.

The risk factors for delayed perforation remain unclear due to the small sample size. In this study, two of the seven patients with delayed perforation had a history of gastric surgery, which was significantly more common than in patients without delayed perforation (*p* = 0.016). One patient underwent gastric tube placement, whereas the other underwent Billroth I reconstruction. Suzuki et al. [16] identified a significant correlation between the presence of a gastric tube and delayed perforation. Nonaka et al. [21] suggested that impaired blood supply to the gastric tube could hinder the healing of post-ESD ulcers, leading to delayed perforation. Yabuuchi et al. [22] reported a 1.3% rate of delayed perforation in the remnant stomach, which was higher than that in normal stomachs, with this possibly being because bile reflux affects ulcer healing. Patients with delayed perforations also show a higher rate of histopathological ulceration than those without them. Histopathological ulceration often indicates submucosal fibrosis, which hinders adequate lifting during submucosal injections in ESD. Therefore, cutting near the muscle layer can cause excessive thermal damage and contribute to a delayed perforation. Hatta et al. [23] reported that closing ESD-induced ulcers could prevent delayed perforation when there is significant thermal damage to the muscle layer. Additionally, although risk factors for intraoperative perforation include a large diameter, lesions in the U-region, ulcer scars, and a location on the greater curvature [5,6], this study found no significant difference between delayed and intraoperative perforations except for that of a higher rate of postoperative stomach conditions in delayed perforation cases. A high odds ratio of 20.45 suggests that postoperative stomach conditions are a characteristic risk factor for delayed perforation. The lack of significant differences could be attributed to small sample sizes.

In this study, two patients with localized peritoneal irritation symptoms were managed conservatively to avoid emergency surgery. One patient underwent endoscopic closure with clips, whereas the other underwent fasting and gastric tube placement. In both the patients, CT only revealed the presence of free air and no perigastric fluid collection. Patients with delayed perforation who avoided emergency surgery tended to exhibit an earlier onset of symptoms such as abdominal pain and fever. The early onset of symptoms facilitated the diagnosis of delayed perforation before oral intake, thereby potentially preventing the exacerbation of peritonitis caused by a leakage of gastric contents.

In this study, second-look endoscopy was performed on three of five patients who underwent emergency surgery. Second-look endoscopy has traditionally been used to prevent postoperative bleeding after gastric ESD. However, two patients with delayed perforation who avoided emergency surgery did not undergo a second-look endoscopy. Among the three patients who underwent second-look endoscopy, two underwent additional hemostasis on the exposed blood vessels of the post-gastric ESD ulcer using coagulation forceps. Additionally, one of the two patients developed postoperative bleeding after second-look endoscopy, and hemostasis was achieved using coagulation forceps. As mentioned previously, the primary causes of delayed perforation are tissue ischemia and necrosis resulting from excessive energization of the muscle layer. In both cases, excessive coagulation in the muscle layer appeared to have caused delayed perforation. Second-look endoscopy reportedly does not prevent postoperative bleeding after gastric ESD [24]. Furthermore, hemostasis using coagulation forceps during second-look endoscopy may lead to delayed perforation. From the above, it is assumed that a second-look endoscopy following gastric ESD is unlikely to be necessary.

Here, we describe a case of delayed perforation after gastric ESD. Although delayed perforation is a serious complication that necessitates emergency surgery, some cases could be mitigated with conservative treatment. Emergency surgery imposes a significant physical burden on patients. Therefore, it would be beneficial if their condition could be alleviated with conservative treatment. However, if this condition necessitates emergency surgery, it should be performed without hesitation. The key is to collaborate closely with the surgeon and monitor the patient if a delayed perforation occurs.

This study has limitations including its retrospective nature and being conducted at a single tertiary referral center. Additionally, the limited sample size requires large-scale studies.

Delayed perforation is a significant complication requiring careful monitoring, particularly in patients with postoperative gastric conditions. The early detection of symptoms such as abdominal pain and fever is essential to prevent serious outcomes such as emergency surgery.

## Figures and Tables

**Table 1:** Characteristics of delayed perforation cases.

Characteristic	Delayed Perforation *n* = 7
Age (years), Median (IQR)	74 (66~79)
<75	4 (57.1)
≧75	3 (42.9)
Sex	
Male	6 (85.7)
Female	1 (14.3)
Hypertension	
No	3 (42.9)
Yes	4 (57.1)
Diabetes mellitus	
No	7 (100)
Yes	0 (0)
Tumor location	
Upper third	1 (14.3)
Middle third	0 (0)
Lower third	6 (85.7)
Postoperative stomach	
Normal stomach	5 (71.4)
Postoperative stomach	2 (28.6)
En bloc resection	
En bloc resection	7 (100)
Partial resection	0 (0)
En bloc complete resection	
En bloc complete resection	5 (71.4)
Non-en bloc complete resection	2 (28.6)
Curative resection	
Curative resection	5 (71.4)
Non-curative resection	2 (28.6)
Resection size (mm), median (IQR)	42 (38~55)
<45	4 (57.1)
≧45	3 (42.9)
Tumor size (mm), median (IQR)	17 (10~22)
<20	5 (71.4)
≧20	2 (28.6)
Tumor shape (endoscopy)	
0-I	1 (14.3)
0-IIa	3 (42.8)
0-IIb	1 (14.3)
0-IIc	2 (28.6)
Combined	0 (0)
Tumor depth	
M	7 (100)
SM1	0 (0)
SM2	0 (0)
Ulceration	
Absent	3 (42.9)
Present	4 (57.1)
Second-look endoscopy performed	3 (42.9)
Symptoms of delayed perforation	
Abdominal pain	6 (85.7)
Fever	1 (14.3)
Symptom onset time (h), median (IQR)	14.4 (5.3–17.8)
Time to diagnosis (h), median (IQR)	23 (11–183.4)
CT findings	
Free air	6 (85.7)
Fluid collection	1 (14.3)
Other complications	
None	6 (85.7)
Delayed bleeding	1 (14.3)
Treatment	
Conservative treatment	2 (28.6)
Surgery	5 (71.4)

Data were presented as the unweighted number (percentage) of patients unless otherwise indicated. Abbreviations: IQR, interquartile range; CT, computed tomography.

**Table 2:** Comparison of cases of delayed perforation and those without delayed perforation.

Characteristic	Delayed Perforation *n* = 7	Without Delayed Perforation *n* = 706	*p* Value
Age (years), median (IQR)	74 (66–79)	74 (67–80)	0.764
<75	4 (57.1)	370 (52.4)	
≧75	3 (42.9)	336 (47.6)	
Sex			1.000
Male	6 (85.7)	541 (76.6)	
Female	1 (14.3)	165 (23.4)	
Hypertension			1.000
No	3 (42.9)	296 (41.9)	
Yes	4 (57.1)	410 (58.1)	
Diabetes mellitus			0.362
No	7 (100)	541 (76.6)	
Yes	0 (0)	165 (23.4)	
Tumor location *			0.842
Upper third	1 (14.3)	128 (16.8)	
Middle third	0 (0)	105 (13.8)	
Lower third	6 (85.7)	529 (69.4)	
Postoperative stomach *			0.016
Normal stomach	5 (71.4)	741 (97.2)	
Postoperative stomach	2 (28.6)	21 (2.8)	
En bloc resection *			1.000
En bloc resection	7 (100)	760 (99.7)	
Partial resection	0 (0)	2 (0.03)	
En bloc complete resection *			0.043
En bloc complete resection	5 (71.4)	726 (95.3)	
Non-en bloc complete resection	2 (28.6)	36 (4.7)	
Curative resection *			0.224
Curative resection	5 (71.4)	665 (87.3)	
Non-curative resection	2 (28.6)	97 (12.7)	
Resection size (mm), median (IQR) *	42 (38–55)	44 (35–55)	0.661
<45	4 (57.1)	380 (51.1)	
≧45	3 (42.9)	372 (48.9)	
Tumor size (mm), median (IQR) *	17 (10–22)	15 (10–22)	0.711
<20	5 (71.4)	500 (65.6)	
≧20	2 (28.6)	262 (34.4)	
Tumor shape (endoscopy) *			0.092
0-I	1 (14.3)	19 (2.5)	
0-IIa	3 (42.9)	233 (30.6)	
0-IIb	1 (14.3)	31 (4.1)	
0-IIc	2 (28.5)	433 (56.8)	
Combined	0 (0)	46 (6.0)	
Tumor depth *			1.000
M	7 (100)	673 (88.3)	
SM1	0 (0)	53 (7.0)	
SM2	0 (0)	36 (4.7)	
Ulceration *			0.026
Absent	3 (42.9)	622 (81.6)	
Present	4 (57.1)	140 (18.4)	

* A total of 7 lesions with delayed perforation and 762 without delayed perforation were analyzed. Data were presented as the unweighted number (percentage) of patients unless otherwise indicated. Abbreviations: IQR, interquartile range.

**Table 3:** Multivariate analysis of factors associated with delayed perforation.

Characteristics	Univariate Analysis			Multivariate Analysis		
	Odds Ratio	95% CI	*p* Value	Odds Ratio	95% CI	*p* Value
Age (years), median (IQR)						
<75	Reference					
≧75	0.83	0.18–3.72	0.803			
Sex						
Male	Reference					
Female	0.55	0.07–4.57	0.577			
Postoperative stomach						
Normal stomach	Reference			Reference		
Postoperative stomach	14.11	2.59–76.97	0.002	23.1	3.59–148.64	0.001
Ulceration						
Absent	Reference			Reference		
Present	5.92	1.31–26.8	0.021	8.37	1.64–42.7	0.016

Logistic regression method was used. Abbreviations: CI, confidence interval.

## Data Availability

The data presented in this study are available upon reasonable request from the corresponding authors. Data were not publicly available because of the inclusion of information that could compromise the privacy of the research participants.

## Supplementary Materials

The following supporting information can be downloaded at: https://www.mdpi.com/article/10.3390/jcm13051317/s1, Table S1. Comparison of delayed perforation and intraoperative perforation cases. Table S2. Comparison of surgery and without surgery cases.
