# Supplementary material for: Characteristics and Risk Factors of Delayed Perforation in Endoscopic Submucosal Dissection for Early Gastric Cancer
Source: J Clin Med. 2024 Feb 26;13(5):1317. doi: 10.3390/jcm13051317 (PMC10931556; doi:10.3390/jcm13051317)
Supplement: Supplementary file 1 [file jcm-13-01317-s001.zip › jcm-2863048-supplementary.pdf]

Supplemental Table S1. Comparison of delayed perforation and intraoperative perforation cases

Characteristics	Delayed perforation n=7	Intraoperative perforation n=22	Odds ratio	95% CI	*p* Value
Age (years), Median (IQR)	74 (66-79)	78 (67.25-83)			
<75	4 (57.1)	15 (68.2)			
≧75	3 (42.9)	7 (31.8)	1.61	0.28-9.20	0.665
Sex					
Male	6 (85.7)	4 (18.2)	Reference		
Female	1 (14.3)	18 (81.8)	0.75	0.07-8.09	1.000
Hypertension					
No	3 (42.9)	12 (54.6)	Reference		
Yes	4 (57.1)	10 (45.5)	1.6	0.29-8.90	0.682
Diabetes mellitus					
No	7 (100)	19 (86.4)	Reference		
Yes	0 (0)	3 (13.6)	0.37	0.02-8.09	0.558
Tumor location*					
Upper third	1 (14.3)	8 (36.4)	Reference		
Middle third	0 (0)	4 (18.2)	0.63	0.02-18.84	1.000
Lower third	6 (85.7)	10 (45.5)	4.8	0.48-48.46	0.355
Postoperative stomach*					
Normal stomach	5 (71.4)	22 (100)	Reference		
Postoperative stomach	2 (28.6)	0 (2.8)	20.45	0.85-490	0.052
En bloc resection*					
En bloc resection	7 (100)	20 (90.9)	Reference		
Partial resection	0 (0)	2 (9.1)	0.55	0.02-12.75	1.000
En bloc complete resection*					
En bloc complete resection	5 (71.4)	20 (90.9)	Reference		
Non en bloc complete resection	2 (28.6)	2 (9.1)	4	0.45-35.79	0.215
Curative resection*					
Curative resection	5 (71.4)	18 (81.8)	Reference		
Non curative resection	2 (28.6)	4 (18.2)	1.8	0.25-12.85	0.558
Resection size (mm), Median (IQR)*†	42 (38-55)	50 (35.25-59.5)			
<45	4 (57.1)	9 (45.0)	Reference		
≧45	3 (42.9)	11 (55.0)	0.614	0.11-3.49	0.678
Tumor size (mm), Median (IQR)*‡	17 (10-22)	15 (13-35)			
<20	5 (71.4)	14 (66.7)	Reference		
≧20	2 (28.6)	7 (33.3)	0.8	0.12-5.21	1.000
Tumor shape (endoscopy)*					
0-I	1 (14.3)	2 (9.1)	Reference		
0-IIa	3 (42.9)	7 (31.8)	0.86	0.05-13.48	1.000
0-IIb	1 (14.3)	0 (0)	5.00	0.11-220	1.000
0-IIc	2 (28.5)	12 (54.5)	0.33	0.02-5.64	0.464
Combined	0 (0)	1 (4.6)	0.56	0.01-24.52	1.000
Tumor depth*					
M	7 (100)	19 (86.4)	Reference		
SM1	0 (0)	1 (4.5)	0.87	0.03-23.72	1.000
SM2	0 (0)	2 (9.1)	0.52	0.02-12.15	1.000
Ulceration*					
Absent	3 (42.9)	14 (63.6)	Reference		
Present	4 (57.1)	8 (36.4)	2.33	0.41-13.17	0.337

* Seven lesions with delayed perforation and 22 lesions with intraoperative perforation were analyzed.

†Two lesions were observed in which the resection size was unknown owing to the failure of en bloc resection in the intraoperative perforation case.
‡One lesion was observed in which the tumor size was unknown owing to the failure of en bloc resection in the intraoperative perforation case.
Data were presented as unweighted number (percentage) of patients unless otherwise indicated.
Abbreviations: IQR, interquartile range

Supplemental Table S2. Comparison of surgery and without surgery cases

Characteristic	Surgery n=5	Without surgery n=2	*p* Value
Age (year), Median (IQR)	71 (66-76)	74 (64.5-80.5)	0.847
<75	3 (60)	1 (50)	
≧75	2 (40)	1 (50)	
Sex			0.286
Male	5 (100)	1 (50)	
Female	0 (0)	1 (50)	
Hypertension			0.143
No	1 (20)	0 (0)	
Yes	4 (80)	2 (100)	
Diabetes mellitus			1.000
No	0 (0)	0 (0)	
Yes	5 (100)	2 (100)	
Tumor location			1.000
Upper third	1 (20)	0 (0)	
Middle third	0 (0)	0 (0)	
Lower third	4 (80)	2 (100)	
Postoperative stomach			1.000
Normal stomach	3 (60)	2 (100)	
Postoperative stomach	2 (40)	0 (0)	
En bloc resection			1.000
En bloc resection	5 (100)	2 (100)	
Partial resection	0 (0)	0 (0)	
En bloc complete resection			1.000
En bloc complete resection	4 (80)	1 (50)	
Non en bloc complete resection	1 (20)	1 (50)	
Curative resection			1.000
Curative resection	4 (80)	1 (50)	
Non curative resection	1 (20)	1 (50)	
Resection size (mm), Median (IQR)	45 (39.5-82.5)	39 (38-40)	0.333
<45	2 (40)	2 (100)	
≧45	3 (60)	0 (0)	
Tumor size (mm), Median (IQR)	18 (11-51)	12.5 (8-17)	0.333
<20	3 (60)	0 (0)	
≧20	2 (40)	2 (100)	
Tumor shape (endoscopy)			0.095
0-I	0 (0)	1 (50)	
0-IIa	3 (60)	0 (0)	
0-IIb	0 (0)	1 (50)	
0-IIc	2 (40)	0 (0)	
Combined	0 (0)	0 (0)	
Tumor depth			1.000
M	5 (100)	2 (100)	
SM1	0 (0)	0 (0)	
SM2	0 (0)	0 (0)	
Ulceration			0.429
Absent	3 (60)	0 (0)	
Present	2 (40)	2 (100)	
Second-look endoscopy performed	3 (60)	0 (0)	0.429
Symptom onset time (hr), Median (IQR)	15.3 (11.7-166.6)	4.05 (2.8-5.3)	0.081
Time to diagnosis (hr), Median (IQR)	27.9 (16-249.7)	16.6 (11-22.2)	0.333

Data were presented as unweighted number (percentage) of patients unless otherwise indicated.
Abbreviations: IQR, interquartile range
